# Public opinion regarding government response to COVID-19: case study of a large commercial city in Nigeria

**DOI:** 10.11604/pamj.2021.38.282.26361

**Published:** 2021-03-17

**Authors:** Ismaeel Yunusa, Sorochi Iloanusi, Osaro Mgbere, Nchebe-Jah Raymond Iloanusi, Anthony Idowu Ajayi, Ekere James Essien

**Affiliations:** 1 Department of Clinical Pharmacy and Outcomes Sciences, University of South Carolina College of Pharmacy, Columbia, South Carolina, USA,; 2 Department of Pharmaceutical Health Outcomes and Policy, University of Houston College of Pharmacy, Houston, Texas, USA,; 3 Institute of Community Health, University of Houston College of Pharmacy, Houston, Texas, USA,; 4 General Hospital, Onitsha, Anambra State, Nigeria,; 5 Population Dynamics and Sexual and Reproductive Health and Rights Unit, African Population and Health Research Center, African Population and Health Research Center (APHRC) Campus, Manga Close, Nairobi, Kenya

**Keywords:** COVID-19, public opinion, government response, knowledge attitude and practices survey, Nigeria

## Abstract

**Introduction:**

government measures to contain the COVID-19 pandemic cannot be effective without widespread compliance by the public. A greater understanding of citizens' perceptions of these measures can help government agencies adapt their strategies to boost compliance. We examined citizens' perceptions of government's measures to contain the COVID-19 pandemic and its implications on compliance using data from Onitsha city, Anambra State Nigeria.

**Methods:**

data was obtained through in-person interviews of 140 consenting adults in March 2020. Descriptive and inferential statistics were used to summarize the data.

**Results:**

most participants (84.7%) doubted government's ability to manage the COVID-19 outbreak, raising concerns about ineffective governance (25.7%) and inadequate health facilities (20.7%). However, participants expressed a favorable perception of school closures (92.3%) and a ban on large gatherings (83.9%), driven mostly by the need to contain the COVID-19 and avoid its spread. But, they were generally indifferent about the closure of the markets and workplaces due to concerns for food insecurity and lack of government's relief programs. Participants who had a positive perception of the ban on large gatherings were more likely to have high knowledge and to adopt good COVID-19 preventive practices.

**Conclusion:**

the study showed a lack of public's confidence in the government's ability to manage the pandemic. This provides an opportunity for the city government and the public to reflect on the existing relationships, build mutual trust, and devise collaborative engagement that will boost compliance and help contain the devastating impact of COVID-19 pandemic.

## Introduction

The high mortality and morbidity rates of coronavirus disease 2019 (COVID-19), combined with its high degree of contagion, have compelled governments worldwide to implement measures to protect citizens from the Severe Acute Respiratory Syndrome Coronavirus 2 (SARS-CoV-2) infection [1]. Currently, there are more than 24 million confirmed cases and over 800,00 deaths worldwide [2]. Governments, via recommendation from International and local public health agencies instituted mandates to mitigate the spread of COVID-19 [3-6]. Mandates, such as state lockdowns, school closures, the shutdown of non-essential businesses, social distancing, and facial masks, are effective ways to reduce new infections [7, 8]. However, they are incredibly disruptive to businesses, the economy, education, government services, and everyday life [9, 10]. Hardships and loss caused by these measures and by the illness create societal tensions and raise questions among the public about the wisdom, effectiveness, and trustworthiness of those in power [11]. Where countries have taken rapid and potent steps to fight against the pandemic, they have also faced resistance and non-compliance [12, 13].

Previous research across Nigeria have assessed the knowledge, attitude and practices (KAP) of the public towards COVID-19 [14-24], but studies on perceptions of government´s measures to curtail its spread are limited, especially in Nigeria. To control the spread of the SARS-CoV-2, the Nigerian government, like many others, imposed a state lockdown, social distancing, self-quarantine for those with any flu-like symptoms, school closure, and shutting down of non-essential shops, supermarkets, and businesses. These measures are drastic and new, with untoward hardship and devastating consequences on people, yet necessary to prevent the unmitigated spread of this highly infectious and deadly disease. This raises a critical question of how people view these measures and the impact of their perceptions on their compliance. Such feedback and reviews from the Nigerian people would help government agencies adapt their strategies to boost compliance with measures designed to limit the spread of the outbreak. Our study examines the public perceptions of the government's measures in response to the COVID-19 pandemic using data obtained from adults in Onitsha, the commercial hub of South-Eastern Nigeria. Understanding the perceptions of adults in a densely populated city like Onisha is critical for government agencies to adapt their strategies to boost compliance.

## Methods

### Study design and participants

We conducted a secondary analysis of a cross-sectional data obtained from a KAP survey in Onitsha, Anambra State, Nigeria in the March 2020 period of the pandemic before the government mandated lockdown on March 29, 2020 [25]. A convenience sampling method was used to recruit 140 study participants from different representative locations within the city of Onitsha, including the commercial markets and housing units. The survey was conducted through in-person interviews of consenting adults aged 18 years and above living and/or working in Onitsha, a large commercial city in South-Eastern Nigeria. More detailed description of the survey instrument used, data collection procedures and the study area can be found in Iloanusi *et al*. [25].

### Analytical measures

The analytical dataset used captured the demographic characteristics, namely gender (female, male), age group (18-24, 25-34, 35-44, 45-54, and 55^+^ years), educational level (primary education or less, secondary education, diploma/associate degree and bache-lors/postgraduate degree), occupation (civil servant, trader/business owner/self-employed, health care worker, student and other), and number of individuals living in household (1, 2-4, 5-7 and 8^+^ persons). The survey assessed the residents' opinions or perceptions of govern-ment actions following the COVID-19 outbreak in Nigeria. Participants were asked to assess the government's ability to manage the COVID-19 outbreak and their views on the school closures, ban on large gatherings, and markets and work closures. They were expected to indicate if they had a *“positive”* or *“negative”* opinion or perception of the government actions in response to the COVID-19 outbreak. Participants who expressed no opinion (*don't know*) for the government actions were excluded from the opinion analyses. In addition, participants were asked to give reasons for their choice of opinions on government actions. Using a text ex-plorer (SAS Institute Inc. 2020. JMP® 14 Text Explorer. Cary, NC, USA: SAS Institute Inc.), the reasons given were parsed and later categorized as follows: Government ability to manage COVID-19 outbreak (*'Ineffective government', 'inadequate health facilities', 'other reason'*and *'no reason given'*); School Closure (*'To avoid COVID-19 spread', 'other reason'*and *'no reason given'*); Ban on large gatherings ('To avoid COVID-19 spread', 'belief in divine intervention', other reason' and 'no reason given') and Markets and work closures (*'Food insecurity', 'to avoid COVID-19 spread', 'lack of govern-ment relief programs', 'financial insecurity' and 'no reason given'*). The residents' knowledge-base of COVID-19, attitude toward COVID-19 management and actual adoption of prevention practices (i.e. KAP) were assessed following previous study [25] classification levels as “low” or “high” for COVID-19 related knowledge and “poor” or “good” for attitude and prevention prac-tices, respectively. Prevention practices were based on the national guidelines for COVID-19 as recommended by the Nigeria Center for Disease Control (NCDC) [5].

### Data analysis

Descriptive statistics were used to outline the demographic characteristics of the sample popula-tion. Univariable analyses of the participants' perceptions of government actions and the reasons for their choice of opinion were carried out using the chi-square tests. Furthermore, we performed bivariate chi-square tests to determine the independent associations between the residents' per-ceptions of government ability and response efforts, and their compliance. To evaluate the impact of the residents' opinions on KAP, we conducted multivariable logistic regression model analyses using residents' opinion on government ability to contain COVID-19 outbreak and government actions (school closures, ban on large gatherings, and markets and work closures) to prevent and control COVID-19 as independent covariates. All independent covariates were included in the KAP models without any condition. We used *“negative”* opinion as referent for all co-variates. The level classifications *“high”* or *“good”*, represented as “1” were used as dependent target (event) and *“low”* or *“poor”* represented as “0” were used as referent for knowledge, and attitude and prevention practice, respectively. We computed both the unadjusted odds ratio (uaOR) and adjusted odds ratio (aOR) along with the 95% confident intervals (CI) and corresponding p-values for each factor within knowledge, attitude, and pre-vention practice models. All statistical tests performed were 2-tailed, with a probability value of 0.05 used as the threshold for declaring statistical significance. Data management and statistical analyses were conducted using SAS JMP Statistical DiscoveryTM Software version 14.3 (SAS Institute, Cary, North Carolina, USA).

### Human subject protection

All relevant ethical guidelines including Institutional Review Board approval process were followed in the conduct of the primary data collection [25]. The current study protocol was reviewed and approved by the Institutional Review Board of the University of Houston, Houston, Texas, USA.

## Results

### Characteristics of study population

**Figure 1:**
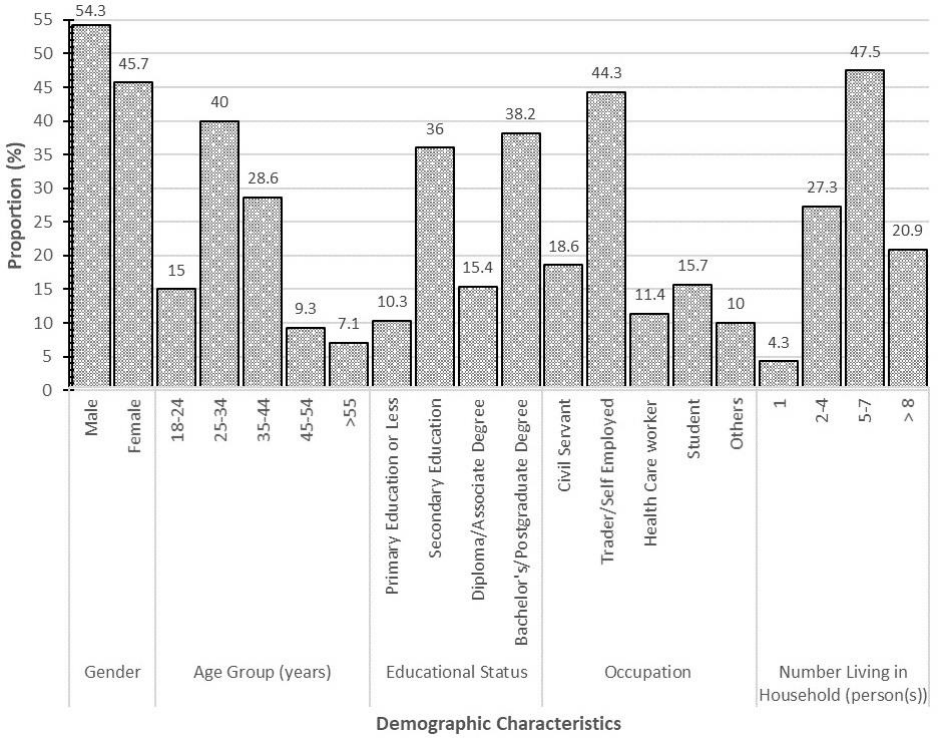
baseline characteristics

The demographic characteristics of the study population is presented in Figure 1. This has been described in detail elsewhere [25]. In summary, the average age of the participants was 34.5 (Standard Deviation (SD): ± 10.9) years. Over half of respondents were males (54.3%) and 38.2% held a bachelor's degree or higher. The occupation of majority of the participants was trading/business ownership (44.3%) and most (47.5%) lived in households with 5-7 persons.

### Peoples' perceptions of government's actions to prevent and control COVID-19

The perceptions of the participants towards government´s actions to prevent and control COVID-19 are shown in Table 1. Although the majority of the participants had a negative perception of the government's ability to manage the COVID-19 outbreak (84.74%, p < 0.001), they had positive views of government´s decision to close schools (92.31%, p < 0.0001) and ban large gatherings (83.90% p < 0.0001). Nevertheless, the city residents were indifferent (p = 0.7718) about the government closure of markets and workplaces due to the threats of COVID-19 pandemic.

**Table 1:** peoples' perception of government's actions to prevent and control COVID-19 in Onitsha City, Nigeria

Government action	Citizens' perception			
	Positive n (%)	Negative n (%)	X^2^-value	P-value
Government ability to manage COVID-19 outbreak	14 (15.22)	78 (84.78)	44.52	<0.0001****
School closures	108 (92.31)	9 (7.69)	83.77	<0.0001****
Ban on large gatherings	99 (83.90)	19 (16.10)	54.24	<0.0001****
Markets and work closures	55 (51.40)	52 (48.60)	0.08	0.7718

Significance level: *=p<0.05, **=p<0.01, ***=p<0.001 ****=p<0.0001.

### Reasons for citizens' perceptions of government´s actions to prevent and control COVID-19

Table 2 highlights reasons given for the residents' perceptions of government response to COVID-19. About one third of participants (32.14%-37.86%, depending on the specific focus of the question) did not give any reason for their perception of government's COVID-19 containment efforts. The two main reasons associated with the residents' negative perception towards government's ability to contain COVID-19 were ineffective governance (25.71%) and inadequate health facilities (20.71%). Most participants believed that the government's decision to close schools (61.43%) and ban large gatherings (54.29%) could curtail COVID-19 spread and these beliefs informed their positive perception of the response efforts. The three main reasons alluded for people's divided opinion on the government's action regarding market and work closures were fear of food insecurity (27.34%), belief that it was necessary to curb the spread of COVID-19 (23.74%) and lack of government relief programs (10.79%).

**Table 2:** reasons for citizens' perception towards government response to COVID-19 pandemic

Reasons for response	n	%	X^2^-value	P-value
**Government ability to manage COVID-19 outbreak**				
Ineffective government	36	25.71	15.14	0.0017**
Inadequate health facilities	29	20.71		
Other reason	22	15.71		
No reason given	53	37.86		
**Schools closures**				
To avoid COVID-19 spread	86	61.43	65.20	< 0.0001****
Other reason	8	5.71		
No reason given	46	32.86		
**Ban on large gatherings**				
To avoid COVID-19 spread	76	54.29	77.96	< 0.0001****
Belief in divine intervention	12	8.57		
Other reason	7	5.00		
No reason given	45	32.14		
**Markets and work closures**				
Food insecurity to avoid COVID-19 spread lack of government relief programs financial insecurity no reason given	38 33 15 8 45	27.34 23.74 10.79 5.76 32.37	35.35	< 0.0001****

Within a given characteristic, the percentages may not add up to exactly 100 due to rounding. Significance level: *=p<0.05, **=p<0.01, ****=p<0.0001.

### Association between people's KAP and perceptions of government's COVID-19 response efforts

The association between people's perceptions towards government's response and their KAP is presented in Table 3. A positive perception of the government's decision to close schools and ban large gatherings was significantly (p < 0.05) associated with good KAP. For instance, approximately 65.74% and 73.74% of participants who had a positive perception of school closures and bans on large gatherings had a high knowledge of COVID-19. Similarly, they were equally more likely to have a good attitude (58.33%, p < 0.05 vs. 61.62%, p < 0.05) and good COVID-19 preventive practices (57.41%, p < 0.05 vs. 62.63%, p < 0.001) to avoid contracting the disease. Having a positive opinion of the government's decision on market and work closure was significantly (p < 0.05) associated with good attitude and prevention practices towards COVID-19 management and compliance to recommended guidelines, respectively. Our study found no significant association (p > 0.05) between the participants' perceptions of government closure of markets and workplaces and knowledge of COVID-19.

**Table 3:** associations between perception of government response, and residents' COVID-19 knowledge, attitude and prevention practice (KAP)

Perceptions of government's response	Knowledge				Attitude				Prevention practice			
	Low n (%)	High n (%)	X^2^-value	P-value	Poor n (%)	Good n (%)	X^2^-value	P-value	Poor n (%)	Good n (%)	X^2^_-_value	P-value
**Ability to manage COVID-19 outbreak**												
Negative	32 (41.03)	46 (58.97)			32 (41.03)	46 (58.97)			35 (44.87)	43 (55.13)		
Positive	6 (42.86)	8 (57.14)	0.02	0.8980	4 (28.57)	10 (71.43)	0.77	0.3793	5 (32.71)	9 (64.29)	40.50	0.5245
**Schools closures**												
Negative	7 (77.78)	2 (22.22)			7 (77.78)	2 (22.22)			7 (77.78)	2 (22.22)		
Positive	37 (34.26)	71 (65.74)	6.71	0.0096**	45 (41.67)	63 (58.33)	4.39	0.0362*	46 (42.59)	62 (57.41)	4.15	0.0416*
**Ban on large gatherings**												
Negative	17 (89.47	2 (10.53)			12 (63.16)	7 (36.84)			16 (84.21)	3 (15.79)		
Positive	26 (26.26)	73 (73.74)	27.50	<0.0001****	38 (38.38)	61 (61.62)	4.01	0.0453*	37 (37.37)	62 (62.63)	14.13	0.0002***
**Markets and work closures**												
Negative	27 (51.92)	25 (48.08)			29 (55.77)	23 (44.23)			31 (59.62)	21 (40.38)		
Positive	19 (34.55)	36 (65.45)	3.29	0.0690	18 (32.73)	37 (67.27)	5.76	0.0164*	21 (38.18)	34 (61.82)	4.92	0.0266*

Significance level**:** *=p<0.05, **=p<0.01, ***=p<0.001 ****=p<0.0001.

### Multivariable logistic regression model

The multivariable logistic regression models of COVID-19 related KAP and the residents' perceptions of government actions are presented in Table 4. In general, participants who had a positive perception of Government closure of schools and ban on large gatherings had high knowledge, good attitude, and adopted good COVID-19 prevention practices. However, when the outcome measures were adjusted against the covariates, only participants who had a positive perception of the ban on large gatherings were more likely to have high COVID-19 related knowledge (aOR: 32.34, 95%CI: 2.99-49.69, p < 0.01) than those who had negative feelings. Although, we noticed no significant (p > 0.05) impact of the residents' positive perception of government actions on their attitude towards the disease management following factors adjustment, those who perceived the ban on large gathering as a positive effort were almost seven times (aOR: 6.73, 95%CI: 1.05-42.76, p < 0.05) more likely to adopt good prevention strategies against COVID-19.

**Table 4:** multivariable logistic regression models for COVID-19 related knowledge, attitude and prevention practice, and citizens' perception of government response to COVID-19 pandemic

Perception of government's COVID-19 response	Knowledge		Attitude		Prevention practice	
	uaOR (95% CI)	aOR (95% CI)	uaOR(95% CI)	aOR (95% CI)	uaOR (95% CI)	aOR (95% CI)
**Ability to manage COVID-19 outbreak** negative (ref) positive	1.00 0.93 (0.29-2.93)	1.00 1.69 (0.22-12.80)	1.00 1.74 (0.50-6.04)	1.00 2.09 (0.21-21.15)	1.00 1.47 (0.45-4.77)	1.00 6.03 (0.52-69.78)
**Schools closures** negative (ref) positive	1.00 6.72 (1.33-33.97) **	1.00 1.50 (0.07-31.73)	1.00 4.90 (0.97-24.69)	1.00 €	1.00 4.72 (0.94-23.77) *	1.00 2.20 (0.13-37.84)
**Ban on large gatherings** negative (ref) positive	1.00 23.87 (5.18-110.44) ****	1.00 32.34 (2.99-49.69) **	1.00 2.75 (1.00-7.60) *	1.00 2.89 (0.60-13.85)	1.00 8.94 (2.44-32.75) ***	1.00 6.73 (1.05-42.76) *
**Markets and work closures** negative (ref) positive	1.00 2.05 (0.94-4.45)	1.00 0.54 (0.17-1.73)	1.00 2.59 (1.18-5.68) *	1.00 2.71 (0.91-8.06)	1.00 2.39 (1.10-5.19) *	1.00 2.25 (0.76-6.66)

Abbreviations: uaOR: unadjusted odds ratio; aOR: adjusted odds ratio; 95%CI: 95% confidence interval; Ref: referent. € Inestimable/unstable parameter due to small sample size and/or complete separation. Tests and confidence intervals on odds ratios are Wald based. Significance level**:** *=p<0.05, **=p<0.01, ***=p<0.001 ****=p<0.0001.

## Discussion

COVID-19 pandemic has devastated the economy, bringing untoward hardship to people's way of life, yet the most potent response to curb its spread remains lockdowns, physical distancing, and wearing face masks, in the absence of a vaccine. Trust in the government's ability to manage the pandemic and contain the virus is important to ensure compliance with recommended guidelines. We examined the public's perceptions of government's response to the COVID-19 pandemic in Nigeria. Our analysis shows that most people doubted the Nigerian government's ability to manage the pandemic, mainly due to their concerns about the poor health infrastructure and ineffective governance plaguing the country. Their concerns are legitimate, given the suboptimal health indices of the country [26, 27]. Access to common illnesses and health facility delivery remains low and disproportionately low in the northern region of the country [28]. An outbreak of a highly infectious disease like COVID-19 will no doubt devastate the already overburdened health system [26].

Also, the finding raises serious concerns about the public's lack of trust in both governance capacity and legitimacy overall [29, 30]. Previous studies have highlighted that there is an existential mistrust of government by citizens of countries throughout Africa [31-37]. A study by Afrobarometer shows that only 31% of Nigerians trust in the state, with many wary of corruption and inadequate support for ordinary citizens [37]. The lack of trust in the government could have adverse consequences on the management of COVID-19 in Nigeria. Citizens mistrust of government in Africa3 leads to the spread of conspiracy theories [38]. As seen during the Ebola outbreaks, when many people believed that the disease was a scam created to steal money, such conspiracy is already spreading but with a claim that the virus came to eliminate corrupt political leaders in Nigeria [39]. Mistrust of government can interfere with the battle against disease and may lead to the eruption of violence as occurred during the Ebola outbreak [40, 41]. The importance of citizens' trust in government has been pointed out in a study that shows how a decline in the public's trust can lead to lower rates of compliance with rules and regulations, which has serious ramifications in times of pandemic [42].

Despite the lack of trust in the government's ability to manage the pandemic, most people approve of the school closure and ban on a large gathering as a necessary prerequisite to curbing the spread of the virus. This, of course, is plausible due to fear of the potentially devastating consequences of a major outbreak, and with the weak health system and government to care for everyone. Nevertheless, they were skeptical when it comes to the closure of the markets and workplaces due to concerns for food insecurity and the absence of government relief programs. This underscored the need for the government to have in place policies, strategies, and institutionalized means of ensuring social protection for all, especially the very poor and vulnerable in times of crisis [12]. Interestingly, our findings suggest a link between perceptions of government's responses and COVID-19 related knowledge attitude and practices. It appears the positive perceptions are associated with better compliance with the recommended preventive practices. However, given the study sample size, the confidence intervals are too wide to conclude on these links. As such future studies with a large sample size could explore these links further.

### Policy implications of study findings

Our finding calls for concern as research has shown that government and experts can learn a lot by understanding public concerns, especially when it comes to implementing restrictive measures in low-income regions [43]. The government needs to overcome widespread mistrust in governance to help medical, and public health professionals address population-based inequalities on health outcomes [44]. This will require improving the health infrastructure throughout the country and curbing corruption. Also, the results of our study could be used to improve public health in Nigeria. They might be considered a wake-up call to authorities to reconsider their relationship with citizens and improve overall communication and trust. It could be used to create effective governance and include real residents in the decision-making process to control the current pandemic and create a solid ground for future ones.

### Limitations

This study has several limitations. First, given the study's small sample, the relationship between perceptions of response measures and KAP yielded wide confidence intervals, suggesting unreliable estimates. Also, the study's small size and convenience sampling mean the findings are not generalizable. The findings of this study and its generalizability should be understood in the context of these limitations. Nevertheless, the study provides early data on public perceptions of government response to COVID-19 and trust in the state's ability to manage the pandemic.

## Conclusion

The study showed a lack of public's confidence in the government's ability to manage the pandemic. Despite this, the positive perceptions toward some governmental actions among the city residents appear to be associated with better compliance with the recommended preventive practices. This provides an opportunity for the city government and the public to reflect on the existing relationships, build mutual trust, and devise collaborative engagement that will boost compliance and help contain the devastating impact of COVID-19 pandemic. Additional studies might be useful to fully understand the issues with the people-governance relationship behind these results.

### What is known about this topic

- To control the spread of the COVID-19 pandemic, the Nigerian government, like many oth-ers, imposed a state lockdown, social distancing, self-quarantine for those with any flu-like symp-toms, school closure, and shutting down of non-essential shops, supermarkets, and businesses;
- Most people approve of school closure and ban on a large gathering as a prerequisite to curb-ing the spread of the pandemic;
- Trust in the government's ability to manage the pandemic and contain the virus is essential to ensure compliance with recommended guidelines.

### What this study adds

- This study provides evidence on how the public views government mandates to control COVID-19 and the impact of their perceptions on their compliance;
- This study showed a lack of public confidence in the government's ability to manage the spread of the COVID-19 pandemic.
